# The Pathogenesis of Quinine Pneumonic Emphysema1Presented at the Thirty-ninth Annual Meeting of the American Veterinary Medical Association, Minneapolis, Minn., September 2, 3, 4, and 5, 1902.

**Published:** 1902-09

**Authors:** A. H. Baker

**Affiliations:** Chicago, Ill.


### THE PATHOGENESIS OF EQUINE PNEUMONIC EMPHYSEMA.[1]

By A. H. Baker, V.S.,
CHICAGO, ILL.

Considering the fact that this disease materially injures the victim of it, reducing his market value and usefulness—in some cases almost to uselessness—and considering that it is very prevalent in the eastern and increasing in the western part of this

[1] Presented at the Thirty-ninth Annual Meeting of the American Veterinary Medical Association, Minneapolis, Minn., September 2, 3, 4, and 5, 1902.

country, it becomes one of the most important subjects for our consideration and our duty to exert our influence on the farmers, to induce them to harvest their fodder at such a time as to reduce the indigestibility of it, and on those having the care of horses to observe better hygiene in the feeding and watering of them to prevent this distressing, and in all chronic cases, incurable affection of horses.

Pneumonic emphysema is a reflex neurosis due to faulty dietetics in 99 per cent. of the cases, the remaining 1 per cent. being due to previously existing pulmonary affection, viz., bronchitis. The origin of this reflex neurosis is in the gastric ramifications of the pneumogastric or vagus nerve. This neurosis, arising in the stomach, produces morbid anatomy in the lungs primarily and only; the cardiac complication arises secondarily and consequently. The gastric manifestations are entirely functional, those of indigestion, among which I may mention thinness of flesh, pot-belly, long rough coat, profuse evacuation of gas per ano, and the liability to flatulent colic, owing to the distention of the stomach and reduction in quantity of the gastric juice. The bowels also become greatly distended, and digestion is so tardy and weak that fermentation of the food is excessive, giving rise to the increased flatus. The disease is induced by a gluttonous appetite; a poor feeder never has it. The horse with heaves is inclined to eat everything in sight. It is produced by long-continued over-eating of over-ripe hay. The two kinds of hay that I have had experience with as causing emphysema are timothy and red clover. These grasses when left to get too nearly ripe before being cut for hay become very ligneous in their stalks, so much so that the lower half of each stalk is wholly unfit for food for horses. This over-ripe hay fed liberally to horses for weeks or months produces a long-continued over-distention of the stomach of the gluttonous feeder; this produces a neurosis of the gastric portion of the vagus, at first hyperæsthesia, followed by enervation, which creeps forward and involves the pulmonary branch, the source of pulmonary enervation, in a neurasthenia that weakens the contractility of the lung substance and lessens its power to expel the tidal air. The disturbance is purely functional at first, manifested by rapid breathing, frequent cough, dilatation of the nostrils, and more or less lassitude. If the cause is not removed in the course of three to six months the contractility of the parenchyma of the lungs becomes impaired, resulting in dilatation of the vesicles, rupture of many into one, and atrophy and pallor of their walls. This pallor is due to anæmia of the distended vesicles, reducing the calibre of the outlet for the blood from the right side of the heart by obliteration, to a greater or less extent, of the capillaries of the pulmonary tissue, leading to dilatation of the right auricle and ventricle. These disturbances are purely functional for two to six months. During this time the disease may be said to be incipient, and is curable under rigid hygienic rules; but if the cause is not removed the morbid anatomy soon becomes chronic and incurable, and gives rise to the double expiratory motion of the organs of expiration. The first half of the expiratory act is performed naturally and with ease, but the contractility of the lungs is so seriously impaired that the abdominal muscles are brought into strong contraction to expel the balance of the tidal air. When this is accomplished they relax quickly and drop back spontaneously into their natural positions; then the inspiration takes place normally. This disease is subject to frequent exacerbations of symptoms by over-eating and drinking, or active work soon after eating, especially if facing the wind.

It may be safely said that pneumonic emphysema never occurs in horses at pasture, nor on prairie hay, nor in horses getting liberal quantities of oats and correspondingly small quantities of hay, nor on timothy and clover that were cut at the proper time.

After thus considering the connection between cause and effect, the question of prophylaxis naturally arises. The question when is the proper time to cut timothy and clover suggests itself. In my opinion it is when the seed is in the milk. In order to catch them in this stage of development they must be cut a day or two after they are in full bloom. Those grasses mature very rapidly, and once they have passed the full-bloom period the stalks become woody very quickly, so much so that if left a week longer, the lower half of the stalk is not only unfit for food, but actually injurious to the horse that eats it.

As a further protection against emphysema and to maintain the stomach in a healthy condition, horses should be fed sufficient grain to make it unnecessary to give them more than a moderate amount of hay, and gluttonous feeders should be muzzled after they have consumed their rations, to prevent them from engorging themselves on their bedding.

The European authors do not recognize the influence of dietetics in the etiology of this disease. The English hint at it, but fail to give it any importance. Professor Law, in volume i. of his admirable work on *Veterinary Medicine*, on page 274, says under

Causes: "This disease is essentially the result of faulty feeding and working, though pre-existing diseases of the air-passages and sudden, violent, muscular efforts no doubt occasionally contribute to its development." On page 278 he says: "The question arises how a disturbing cause operating directly upon the digestive organs should affect the respiratory in such a marked and permanent manner. It cannot be because of the gastric and abdominal distention, since pregnant mares, though in a state of much greater plenitude, are not thereby rendered liable to broken wind; and if they have previously suffered from this infirmity, the symptoms are less marked when breeding." Then he refers to a theory advanced by Dupuy, that certain disturbances of the stomach and intestines so impair the function of the vagus nerve that the lungs are affected, at first functionally and afterward structurally, but no theory is advanced as to the cause of this disturbance resulting in emphysema. The point I wish to emphasize is that long-continued distention of the stomach, not by any particular kind of food, but by food of a particular quality, is the cause of emphysema, and this particular quality is found in the rank growth of the grasses above mentioned on the rich soil of our Western prairies, in which case it is specially necessary that it should be cut before the stalks become woody.

